# Evaluation of the soft tissue facial profile in different skeletal malocclusions in relation to age

**DOI:** 10.1186/s12903-024-04486-1

**Published:** 2024-06-20

**Authors:** Michał Kiełczykowski, Konrad Perkowski, Konrad Kamiński, Małgorzata Zadurska, Ewa Czochrowska

**Affiliations:** Department of Orthodontics, Medical University in Warsaw, Warsaw, 02-097 Poland

**Keywords:** Camouflage treatment, Cephalometric analysis, Profile analysis, Skeletal malocclusion, Soft tissue analysis

## Abstract

**Background:**

The aim of the study was to assess the thickness of the soft tissue facial profile (STFP) in relation to the skeletal malocclusion, age and gender.

**Methods:**

All patients, aged 7–35 years, who were seeking orthodontic treatment at the Department of Orthodontics, Medical University of Warsaw between 2019 and 22 were included in the study. All patients had lateral head radiographs taken before the treatment. The cephalometric analysis was performed including the STFP analysis. The patients were allocated to one of six groups based on age and skeletal relations (ANB angle). The minimum number of patients in each group was 60 with equal gender distribution. The STFP analysis included ten linear measurements.

**Results:**

A total of 300 patients were included in the study and allocated to five groups. Group 6 (growing patients with skeletal Class III malocclusion) was not included in the study as it failed to achieve the assumed group size. There were significant differences in the thickness of the STFP in relation to the skeletal malocclusions. Adults with skeletal Class III malocclusion had significantly thicker subnasal soft tissues compared to patients with skeletal Class I and Class II malocclusions. The thickness of the lower lip in patients with Class II skeletal malocclusion was significantly bigger compared to the other groups. Children and adolescents with Class II malocclusions had thicker lower lip in comparison to the group with Class I malocclusion. The majority of the STFP measurements were significantly smaller in children and adolescents compared to adults. The thickness of the STFP in males was significantly bigger in all age groups compared to females.

**Conclusions:**

The thickness of facial soft tissues depends on the patient’s age and gender. The degree of compensation of the skeletal malocclusion in the STFP may be a decisive factor during orthodontic treatment planning regarding a surgical approach or a camouflage treatment of skeletal defects.

## Introduction

Soft tissue facial profile (STFP) is the subject of interest for many specialists from different medical fields. In orthodontics and plastic surgery STFP is important regarding diagnosis and treatment planning [1]. The differences in thickness are used in anthropology to determine the facial appearance of ancient populations and in forensic medicine to identify the deceased on the basis of skeletal traits [2].

Maxillary and mandibular morphology and position as well as incisor position and inclination play an important role regarding facial aesthetics. Also, the thickness of overlying soft tissues, their harmony and proportions in the face, is very important, hence its recent application to different treatments, including plastic surgery, to enhance fullness of the profile, or to camouflage underlying skeletal malocclusions.

Ackerman et al. [3] have proposed a paradigm that now prevails in orthodontics: proper STFP relations constitute the most important objective in orthodontic treatment and their proper proportion in relation to the underlying hard tissues determines optimal aesthetics and treatment stability. Holdaway [4] and later Arnett et al. [5] introduced STFP analysis in orthodontic diagnostics and orthognathic surgery. Awareness of how soft tissue thickness changes relative to age, gender or skeletal malocclusion may provide valuable information during orthodontic treatment of patients with gnathic defects. The existence of compensation regarding the thickness of patient’s soft tissue profile in the insufficient growth zone may to a lesser or greater extent conceal the existence of bone disproportion, in this way improving the harmony of the face in a patient who is affected by malocclusion. The higher the compensation, the less visible the gnathic malocclusion in the patient’s profile. Such a situation may motivate both the patient and the doctor to consider more conservative treatment methods including orthodontic camouflage.

Cephalometric analysis is commonly used in orthodontic diagnosis and treatment planning to analyse the sagittal and vertical skeletal and dental relations. At present, clinicians more and more frequently apply Artificial Intelligence algorithms to conduct cephalometric analysis, which may facilitate their work and save time [6]. Because the outline of the patient’s soft tissue profile is visible on teleradiographs it will be accounted for during cephalometric analysis of hard tissues of the jaws and teeth, without unnecessary exposure of the patient to radiation or additional costs. It matters when the orthodontic treatment is planned since STFP thickness influences the patient’s profile after therapy in view of the intended alterations of tooth positions.

In the literature, there are contradictory results concerning soft tissue thickness in relation to the existing skeletal sagittal malocclusion in adult patients. Several of them seem to confirm compensation of skeletal malocclusion by soft tissues, which means that in patients with Class III skeletal malocclusion the soft tissues of the subnasal and the upper lip areas are thicker, while in patients with skeletal Class II malocclusion the lower lip is thicker [7–11]. In growing patients only one study was conducted, which supported the presence of the soft tissue compensation in this age group [12]. The majority of published studies indicate the presence of sexual dimorphism related to soft tissue thickness in adults, manifested by thicker tissues in adult males than in females [13–16]. In the literature, the same observation relates to children although the number of studies on this subject is limited [17–19]. There have been reports concerning changes in the thickness of facial soft tissues at subsequent stages of growth, increasing with age [20–22]. Few published studies dwell on the same subject in adults, with the available reports indicating a positive correlation between soft tissue thickness and age [23–25]. So far, comparative studies concerning STFP thickness differences between children and adults have not been published.

Due to the discrepancies in the published results and the limited number of scientific reports analysing STFP in growing patients the aim of the present study was to assess and to compare the STFP thickness in relation to the skeletal malocclusion, age and gender.

## Materials and methods

All Caucasian patients aged 7–35 years who were orthodontically treated in the Department of Orthodontics, Medical University in Warsaw between 2019 and 2022 were included in the study. The exclusion criteria were: presence of dentofacial deformities, history of craniofacial trauma, previous orthodontic treatment, previous prosthodontic or surgical treatment including any soft tissue augmentation procedures, incompetent lip closure and poor quality of radiographs.

**Fig. 1:**
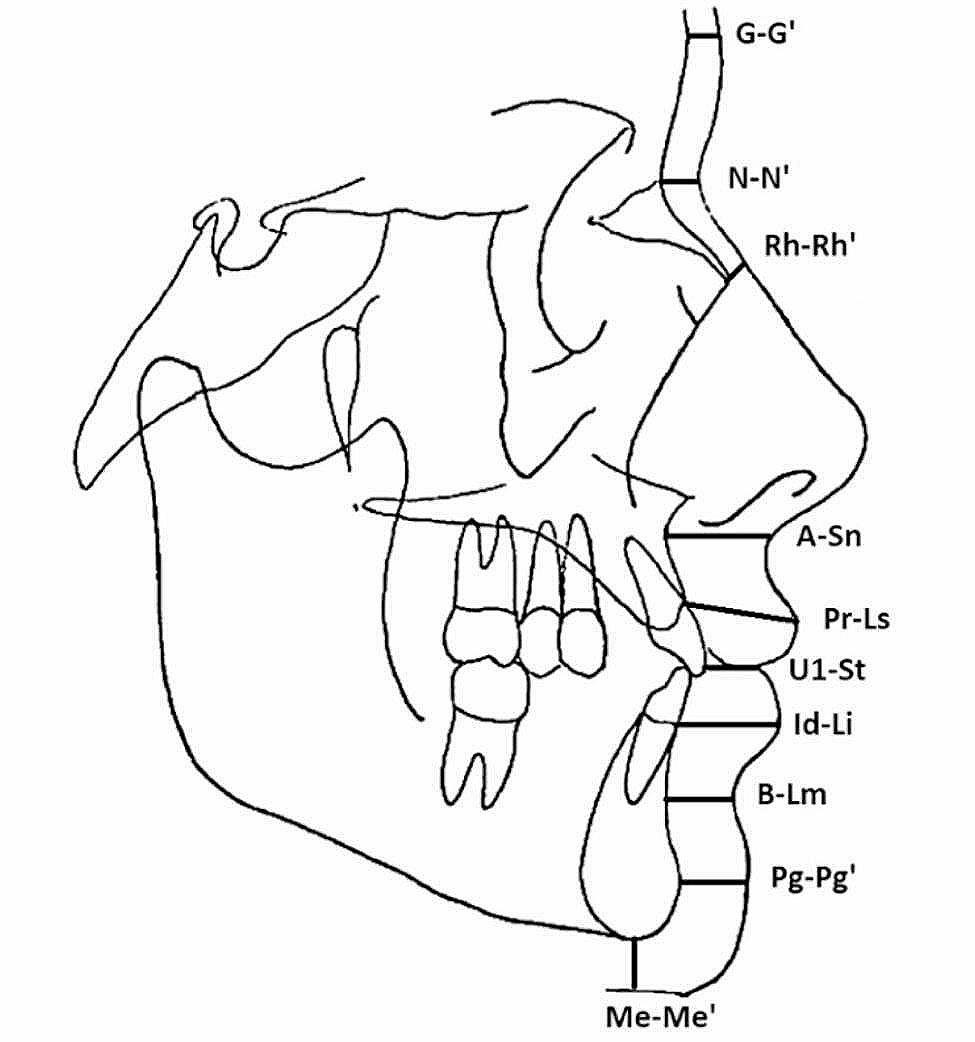
Thickness of the soft tissue profile at landmarks

All the patients had a cephalometric analysis based on the lateral head radiographs taken before the orthodontic treatment. All radiographs were taken with 4-in-1 Dental X-ray Imaging system PAPAYA 3D PLUS. Each radiograph had been calibrated with a millimetre scale before the cephalometric analysis. The cephalometric analysis was performed using the DDP-Ortho 2.10.2022 software (Polorto, Czestochowa, Poland), which was preadjusted for STFP analysis including ten measurements in accordance with the methodology proposed by Utsuno et al., Kamak et al. and Hamid et al. (Table 1; Fig. 1) [7, 8, 17]. The cephalometric analysis was performed by the researcher, who was blinded to the patient’s clinical records and treatment (M.K.).

**Table 1:** Cephalometric analysis of the thickness of the soft tissues facial profile. The ANB angle is in degrees, other measurements in mm

Measurement	Definition
ANB	The angle formed between the Nasion point (the most anterior point on the frontonasal suture in the midline), Point A (the deepest point on the curved profile of the maxilla between the Anterior Nasal Spine and the alveoral crest) and Point B (the deepest point on the curved profile of the mandible between the chin and alveolar crest). Used to determine the skeletal class.
G-G’	Linear distance from the most prominent point on the frontal bone to the soft tissue prominence on the forehead.
N-N’	Linear distance from the skeletal Nasion to the soft tissue Nasion.
Rh	Perpendicular distance from the intersection of the nasal bone and cartilage to the nasal soft tissue.
Sn-A	Linear distance between the point Subnasale and the A point.
Ls-Pr	Linear distance between the most prominent point of the upper lip and the point Prosthion.
St-U1	Linear distance between the most prominent point of the upper incisor and the point Stomion.
Li-Id	Linear distance between the most prominent point of the lower lip and the point Infradentale.
B-Lm	Linear distance from the point B to the Labiomental sulcus.
Pg-Pg’	Linear distance between the skeletal Pogonion and the soft tissue Pogonion.
Me-Me’	Linear distance between the skeletal Menton and the soft tissue Menton.

Based on the value of the ANB angle, the patients were allocated to one of the six groups in relation to age (Table 2). The value of the ANB angle > 4 degrees was defined as skeletal Class II malocclusion, while 0 < as Class III malocclusion. Class I malocclusion was diagnosed for the values between 0 and 4 degrees.

**Table 2:** Allocation of patients to groups

	Age	ANB value/ skeletal class
Group 1	17–35	0–4° / skeletal Class I
Group 2	17–35	> 4° / skeletal Class II
Group 3	17–35	< 0° / skeletal Class III
Group 4	7–16	0–4° / skeletal Class I
Group 5	7–16	> 4° / skeletal Class II
Group 6	7–16	< 0° / skeletal Class III

The inclusion criteria for the minimum sample size was 60 participants with equal gender distribution in each of the groups based on the study by Kamak & Celikoglou [8]. The study was approved by the Bioethics Committee of the Medical University of Warsaw (AKBE/86/2022).

### Statistical analysis

The statistical analysis was performed using PQStat software version 1.6.8. A level of significance was set at p < 0.05. Due to the lack of knowledge about the mean and standard deviation values of the evaluated parameters for the assessed populations, the Lilliefors test was used to analyze the normality of the distribution. The analysis revealed a lack of conformity to a normal distribution for most of the assessed parameters (age in the adult group, age in the children and adolescents’ group, G-G’, N-N’, Rh-Rh’, A-Sn, Pr-Ls, U1-St, Pg-Pg’, Me-Me’, and ANB), the non-parametric tests were utilized in further analyses (U Mann-Whitney and ANOVA Kruskal-Wallis).

### Reliability of the measurements

Twenty randomly chosen cephalometric radiographs were re-traced by the same researcher (M.K.) at four-weeks interval. The obtained results were then compared with the previous cephalometric analysis. The Interclass Correlation Coefficient was used to determine the correlation between the first and the second measurements.

## Results

The characteristics of the study groups including age differences is presented in Table 3. Group 6 was not included in the study as it failed to achieve the assumed group size. No statistically significant differences were demonstrated for age in children and adolescents; for adults, patients in Group 3 (Class III) were younger than patients in Group 1 (Class I) and Group 2 (Class II), and the difference was statistically significant (Table 4).

**Table 3:** Characteristics of patients in groups. The group 6 was not included as it failed to achieve the assumed group size

	Group size	Male	Female	Age range	Mean age + SD	Age comparison	
						Kruskal-Wallis ANOVA	post-hoc analysis
Group 1	60	30 (50%)	30 (50%)	Min: 17 Max: 35	25.5 ± 5.2	*p* = 0.017	Gr. 3 vs. Gr. 1 (*p* = 0.045) Gr. 3 vs. Gr. 2 (*p* = 0.035)
Group 2	60	30 (50%)	30 (50%)	Min: 17 Max: 35	25.6 ± 5.7		
Group 3	60	30 (50%)	30 (50%)	Min: 17 Max: 34	23.0 ± 5.4		
Group 4	60	30 (50%)	30 (50%)	Min: 7 Max: 16	12.5 ± 2.4	*p* = 0.987	
Group 5	60	30 (50%)	30 (50%)	Min: 8 Max: 16	12.5 ± 1.8		

**Table 4:** Comparisons of the STFP thickness relative to skeletal class in adult males and females (ANOVA Kruskal-Wallis test, post-hoc analysis acc. to Dunn with Bonferroni modification)

	Landmark	Class I vs. Class II	Class I vs. Class III	Class II vs. Class III	Mean thickness (mm)		
					I	II	III
Males	G-G’	N.s.	N.s.	N.s.	6.1	5.7	6.4
	N-N’	N.s.	N.s.	N.s.	6.5	6.2	6.9
	Rh-Rh’	N.s.	N.s.	N.s.	2.8	2.7	2.6
	A-Sn	0.036	N.s.	0.001	17.0	15.9	17.4
	Pr-Ls	N.s.	0.001	0.001	15.0	15.0	17.1
	U1-St	N.s.	0.001	0.001	7.2	6.7	9.5
	Id-Li	N.s.	N.s.	0.026	15.5	16.3	15.2
	B-Lms	N.s.	N.s.	N.s.	11.7	12.1	11.7
	Pg-Pg’	N.s.	N.s.	N.s.	12.2	12.6	11.7
	Me-Me’	N.s.	N.s.	N.s.	8.1	8.5	8.2
Females	G-G’	N.s.	N.s.	N.s.	5.7	5.7	5.9
	N-N’	N.s.	N.s.	N.s.	5.6	5.4	5.7
	Rh-Rh’	N.s.	N.s.	N.s.	2.5	2.2	2.2
	A-Sn	N.s.	0.001	0.001	14.0	13.8	15.7
	Pr-Ls	N.s.	0.004	0.023	12.3	12.3	13.9
	U1-St	N.s.	N.s.	0.001	5.4	4.7	6.3
	Id-Li	0.001	N.s.	0.001	13.1	14.8	13.2
	B-Lms	N.s.	N.s.	N.s.	10.7	11.3	10.8
	Pg-Pg’	N.s.	0.049	N.s.	11.2	11.2	10.2
	Me-Me’	N.s.	N.s.	N.s.	6.8	7.0	6.7

### STFP thickness in relation to the skeletal class

Table 4 presents the mean thickness of STFP and standard deviation for each skeletal class in groups comprising adult males and females. In both analyzed groups, the significant differences were observed in measurements of the chin area and both lips (*p* < 0.05); furthermore, in the female group, significant differences were also observed for the chin. Characteristically for males with Class II, the soft tissue thickness was the smallest in the subnasal area (A-Sn) in comparison with other areas. By analogy, males with Class III had the thickest soft tissues at the upper lip (Pr-Ls) and lip contact (U1-St) areas. Soft tissues of the lower lip (Id-Li) were thicker in males with Class II in comparison with Class III patients. In females, the measurement at the subnasal area (A-Sn) and the upper lip (Pr-Ls) revealed the higher thickness in patients with Class III, while the thickness of the lower lip (Id-Li) was the highest in the Class II malocclusion. Females with Class III had thicker soft tissues at the lip contact area (U1-St) than those with Class II malocclusion. The chin thickness (Pg-Pg) was higher in patients with Class I than in Class III malocclusion.

Table 5 presents mean STFP thickness and standard deviation in relation to the skeletal class in boys and girls. The boys with Class I malocclusion had significantly (p < 0.05) higher measurements at the Glabella (G-G’), Subnasale (A-Sn) and the chin (Me-Me’), in contrary to the thickness of the lower lip (Id-Li) where higher values were found for the boys with Class II malocclusion. In girls with Class I malocclusion higher soft tissue thickness was present at the subnasale (A-Sn), upper lip (Pr-Ls) and lip contact (U1-St) areas. Girls with Class II malocclusion had thicker soft tissues at the lower lip (Id-Li) and the chin (Pg-Pg’) areas.

**Table 5:** Comparisons of STFP thickness in relation to skeletal classes in boys and girls (U Mann-Whitney test)

	Landmark	Class I vs. Class II	Mean thickness (mm)	
			Class I	Class II
Boys	G-G’	0.026	6.1	5.5
	N-N’	N.s.	5.9	5.5
	Rh-Rh’	N.s.	2.3	2.4
	A-Sn	0.006	14.9	13.8
	Pr-Ls	N.s.	13.9	13.6
	U1-St	N.s.	5.7	4.9
	Id-Li	0.001	13.7	15.6
	B-Lms	N.s.	10.9	11.1
	Pg-Pg’	N.s.	10.8	10.1
	Me-Me’	0.021	6.8	6.2
Girls	G-G’	N.s.	5.8	5.4
	N-N’	N.s.	5.2	5.3
	Rh-Rh’	N.s.	2.2	2.2
	A-Sn	0.031	13.8	13.0
	Pr-Ls	0.044	12.8	11.8
	U1-St	0.009	5.0	4.2
	Id-Li	0.006	12.5	13.7
	B-Lms	N.s.	9.8	10.0
	Pg-Pg’	0.040	9.6	10.6
	Me-Me’	N.s.	6.0	6.5

### STFP thickness in relation to age

Table 6 presents differences in the mean thickness of STFP and standard deviation for males and females in both age groups in relation to the skeletal malocclusion. As for males, adults with Class I malocclusion had significantly thicker soft tissues in 9 out of 10 measurements, while in Class II malocclusion significantly thicker soft tissues were present in 7 out of 10 measurements (*p* < 0.05).

**Table 6:** Age-related comparisons of STFP thickness (considering gender and skeletal classes). The U Mann-Whitney test

		Landmarks	Adults vs. children and adolescents	Mean thickness (mm)	
				Children and adolescents	Adults
Males	Class I	G-G’	N.s.	6.1	6.1
		N-N’	0.030	5.9	6.5
		Rh-Rh’	0.005	2.3	2.8
		A-Sn	0.001	14.9	17.0
		Pr-Ls	0.006	13.9	15.0
		U1-St	0.001	5.7	7.2
		Id-Li	0.001	13.7	15.5
		B-Lms	0.026	10.9	11.7
		Pg-Pg’	0.003	10.8	12.2
		Me-Me’	0.001	6.8	8.1
	Class II	G-G’	N.s.	5.5	5.7
		N-N’	0.006	5.5	6.2
		Rh-Rh’	N.s.	2.4	2.7
		A-Sn	0.001	13.8	15.9
		Pr-Ls	0.002	13.6	15.0
		U1-St	0.002	4.9	6.7
		Id-Li	N.s.	15.6	16.3
		B-Lms	0.006	11.1	12.1
		Pg-Pg’	0.001	10.1	12.6
		Me-Me’	0.001	6.2	8.5
Females	Class I	G-G’	N.s.	5.8	5.7
		N-N’	N.s.	5.2	5.6
		Rh-Rh’	N.s.	2.2	2.5
		A-Sn	N.s.	13.8	14.0
		Pr-Ls	N.s.	12.8	12.3
		U1-St	N.s.	5.0	5.4
		Id-Li	N.s.	12.5	13.1
		B-Lms	0.001	9.8	10.7
		Pg-Pg’	0.001	9.6	11.2
		Me-Me’	0.009	6.0	6.8
	Class II	G-G’	N.s.	5.4	5.7
		N-N’	N.s.	5.3	5.4
		Rh-Rh’	N.s.	2.2	2.2
		A-Sn	0.031	13.0	13.8
		Pr-Ls	N.s.	11.8	12.3
		U1-St	N.s.	4.2	4.7
		Id-Li	0.004	13.7	14.8
		B-Lms	0.001	10.0	11.3
		Pg-Pg’	N.s.	10.6	11.2
		Me-Me’	N.s.	6.5	7.0

Significantly thicker soft tissues in adult females in comparison with girls were present for 3 measurements in patients with Class I malocclusion (B-Lms, Pg-Pg’, Me-Me’) and Class II malocclusions (A-Sn, Id-Li, B-Lms).

### STFP thickness in relation to gender

The differences in the STFP thickness between adult females and males are presented in Table 7. Males had significantly thicker STFP than females in all measurements except G-G’ measurement. In growing patients, the same tendency towards increased STFP thickness was observed in boys, however, there were more measurements for which the difference between boys and girls was not statistically significant (Table 8). In patients with Class I malocclusion boys had significantly thicker STFP in seven out of ten landmarks; by analogy, in patients with Class II malocclusion higher STFP thickness was demonstrated in five out of ten measurements in boys.

**Table 7:** Gender-related differences in the STFP thickness in adults (U Mann-Whitney test)

	Landmarks	Descriptive statistics				Gender differences
		Male		Female		
		Mean	S.D	Mean	S.D	*p*-value
Class I	G-G’	6.1	0.9	5.7	1.0	0.129 (N.s)
	N-N’	6.5	0.9	5.6	0.8	0.001
	Rh-Rh’	2.8	0.6	2.5	0.6	0.029
	A-Sn	17.0	1.5	14.0	1.2	0.001
	Pr-Ls	15.0	1.7	12.3	1.4	0.001
	U1-St	7.2	1.6	5.4	1.3	0.001
	Id-Li	15.5	1.6	13.1	1.6	0.001
	B-Lms	11.7	1.3	10.7	1.0	0.001
	Pg-Pg’	12.2	1.6	11.2	1.5	0.013
	Me-Me’	8.1	1.5	6.8	1.3	0.002
Class II	G-G’	5.7	0.8	5.7	0.6	0.853 (N.s)
	N-N’	6.2	0.9	5.4	1.0	0.001
	Rh-Rh’	2.7	0.5	2.2	0.4	0.001
	A-Sn	15.9	1.3	13.8	1.4	0.001
	Pr-Ls	15.0	1.7	12.3	2.1	0.001
	U1-St	6.7	2.2	4.7	1.5	0.001
	Id-Li	16.3	1.4	14.8	1.4	0.001
	B-Lms	12.1	1.0	11.3	1.4	0.028
	Pg-Pg’	12.6	1.8	11.2	1.8	0.007
	Me-Me’	8.5	1.7	7.0	1.5	0.001
Class III	G-G’	6.4	1.0	5.9	1.1	0.069 (N.s)
	N-N’	6.9	1.5	5.7	0.9	0.002
	Rh-Rh’	2.6	0.7	2.2	0.5	0.025
	A-Sn	17.4	1.7	15.7	1.7	0.001
	Pr-Ls	17.1	2.2	13.9	2.0	0.001
	U1-St	9.5	1.9	6.3	1.8	0.001
	Id-Li	15.2	1.8	13.2	1.4	0.001
	B-Lms	11.7	1.0	10.8	1.2	0.007
	Pg-Pg’	11.7	2.5	10.2	1.7	0.021
	Me-Me’	8.2	1.8	6.7	1.6	0.001

**Table 8:** Gender-related differences in the STFP thickness in children and adolescents (U Mann-Whitney test)

	Landmarks	Descriptive statistics				Gender differences
		Male		Female		
		Mean	S.D	Mean	S.D	*p*-value
Class I	G-G’	6.1	1.1	5.8	0.8	0.300 (N.s.)
	N-N’	5.9	0.9	5.2	0.8	0.003
	Rh-Rh’	2.3	0.6	2.2	0.4	0.824 (N.s.)
	A-Sn	14.9	1.5	13.8	1.4	0.006
	Pr-Ls	13.9	1.7	12.8	1.6	0.010
	U1-St	5.7	1.5	5.0	1.3	0.139 (N.s.)
	Id-Li	13.7	1.1	12.5	1.7	0.009
	B-Lms	10.9	1.5	9.8	0.7	0.004
	Pg-Pg’	10.8	1.6	9.6	1.3	0.003
	Me-Me’	6.8	1.2	6.0	0.9	0.007
Class II	G-G’	5.5	1.0	5.4	0.9	0.917 (N.s.)
	N-N’	5.5	1.2	5.3	1.0	0.965 (N.s.)
	Rh-Rh’	2.4	0.5	2.2	0.5	0.052 (N.s.)
	A-Sn	13.8	1.4	13.0	1.4	0.027
	Pr-Ls	13.6	2.0	11.8	1.5	0.001
	U1-St	4.9	1.5	4.2	1.1	0.041
	Id-Li	15.6	1.4	13.7	1.3	0.001
	B-Lms	11.1	1.1	10.0	1.3	0.002
	Pg-Pg’	10.1	1.4	10.6	1.9	0.314 (N.s.)
	Me-Me’	6.2	1.3	6.5	1.4	0.301 (N.s.)

High repeatability of the measurements was found (*p* < 0.001), with ICC values > 0.90 for most of the parameters, with the exception of (U1-St and B-Lms) where the values were 0.89 and 0.87, respectively (Table 9).

**Table 9:** Intraclass Correlation Coefficient which determines absolute concordance between the first and the second measurement

	G-G’	*N*-*N*’	Rh-Rh’	A-Sn	Pr-Ls	U1-St	Id-Li	B-Lms	Pg-Pg’	Me-Me’
ICC	0.95	0.95	0.92	0.94	0.91	0.89	0.96	0.87	0.96	0.97
p (test F)	< 0.001	< 0.001	< 0.001	< 0.001	< 0.001	< 0.001	< 0.001	< 0.001	< 0.001	< 0.001

## Discussion

There are many factors influencing therapeutic options for patients with skeletal malocclusion such as the severity of malocclusion and the patient’s expectations. In severe skeletal malocclusions the combined orthodontic and surgical treatment is often applied. It involves orthognathic surgery which aims to obtain normal skeletal relations of the maxilla and the mandible by a surgical repositioning. Alternatively, the treatment plan may involve orthodontic camouflage of the underlying skeletal malocclusion, which aims to achieve the normal dental relations without changing the morphology and position of the maxilla and the mandible. The orthodontic camouflage will not significantly alter the patient’s face despite improvement of occlusion in contrast to the orthognathic surgery. The thickness of the STFP is important when planning the orthodontic treatment in patients with skeletal malocclusions as such differences may camouflage the existing skeletal discrepancies. It was shown in the present study that differences in the STFP thickness are related to the type of skeletal malocclusion, patient’s age and gender.

In the majority of studies evaluating differences in soft tissue thickness of the facial profile, the number of patients included in the study group ranged from 20 to 77 [7–9, 13, 14]. In the present study the minimum number of participants was 60 in each study group. Kamak & Celikoglu evaluated facial soft tissue thickness among different skeletal malocclusions [8]. They have calculated, that the sample size to detect a clinically meaningful difference required 30 male and 30 female patients in each skeletal group, which was included in the present study.

### STFP thickness in relation to the skeletal class

The increased soft tissue thickness in the subnasal, the upper lip and the lip contact areas in adults with skeletal Class III indicated, that STFP may mask the presence of skeletal disproportion by adjustment of their thickness. By analogy, soft tissue compensation was also demonstrated for the increased lower lip thickness in patients with Class II malocclusion. Similar conclusions were reported in few published reports [7–11, 13, 14, 26, 27].

In the present study, only the growing patients with Class I and II malocclusions were compared. That was related to the insufficient number of recruited children with skeletal Class III malocclusion, which is a much rarer sagittal malocclusion in the population. It was demonstrated, that in growing patients the thickness of the lower lip is higher in patients with Class II than in patients with Class I malocclusion. By analogy, the measurement of thickness at the subnasal area (A-Sn) in both genders had lower values in Class II in comparison with Class I skeletal malocclusions. These differences in children and adolescents in relation to the skeletal class were analogous to the results for adults. The correlation between the STFP and the skeletal malocclusion in growing patients were not often previously reported. Pithon et al. demonstrated that the soft tissue thickness in the lip contact area in boys and girls with skeletal Class III malocclusion was greater than in the Class II. In girls with Class II malocclusion the thickness of the lower lip was greater than in the Class III group. In boys with skeletal Class III malocclusion the chin soft tissues (Pg-Pg’) were significantly thicker in comparison with the Class II. On the other hand, Lopatiene evaluated children aged 14–16 years and did not report differences in the upper lip thickness in relation to the skeletal malocclusion [28].

Patients who had lower thickness of the STFP, especially in areas with skeletal discrepancies in the sagittal dimension, the camouflage treatment can be regarded as a compromise, because despite improvement of occlusion, the patient’s profile will still expose underlying skeletal malocclusion, which may be unsatisfactory to the patient. In such clinical situations, the combined orthodontic-surgical treatment should be considered.

### STFP thickness in relation to age

The comparisons of STFP between the adult and growing patients confirmed their increased thickness in adults. Significant differences, however, were not shown for all the measurements. This may be due to the fact that the participating children and adolescents were mixed in the sense that their craniofacial development was at different stages. At the age of 16, both boys and girls finished the greatest growth associated with the period of adolescence, which may indicate the stabilization of the morphology of the craniofacial structures. We have decided to set the age at 16 years based on the results by Mamandras [29], who evaluated lip thickness from the age of 2 until the age of 18 years. He concluded, that no significant changes were observed after the age of 16. For these reasons, it was decided to define the age of 16 as the boundary age for individuals in the growth period in the conducted study. Cervical vertebrae maturation (CVM) is a valuable method for assessing the craniofacial skeletal maturational stage of an individual. In the present study the CVM method was not used, but it is worthy to consider this method in evaluation of the STFP thickness in the future.

Studies published so far indicated, that the STFP thickness increases with age in growing patients [ [18–22]. Smith et al. demonstrated that soft tissues become thicker with age with the most prominent changes occurring in the nasal and subnasal areas [30]. Jeelani indicated the increase of the STFP thickness in the lower part of the face with age, which was not manifested for the soft tissues at the forehead [31]. The STFP thickness changes in adults were not much reported in the literature. Chen et al. performed a MRI examinations in adults and demonstrated a close association of the STFP thickness in relation to age. The mean STFP thickness increased with age in adult males and females, gradually declining after the age of 60 years. The thickest STFP in male and females was found at the ages of 45–59 and 35–44 years, respectively [23]. A study by Drgáčová et al. in adults aged 21–83 years based on CT scans revealed that STFP increased its thickness with age in both males and females [24]. These results were confirmed by Formby et al. who reported age-related STFP thickness changes in adult patients. The soft tissue thickness of the nose and chin increased with age in males, but decreased in the upper lip; the thickness also decreased slightly in the lower lip. In females, the soft tissue thickness increased at the nose, while decreased at the chin and the upper lip. The lower lip thickness increased only slightly [25]. There is a lack of studies which compare the STFP thickness between adult and growing patients.

### STFP thickness in relation to gender

Different authors demonstrated the presence of sexual dimorphism regarding STFP thickness based on cephalometric radiographs [7–9, 13, 15, 32]. They demonstrated higher values of STFP thickness in most of the measurements in males in comparison to females. Inconclusive results were presented by Perović et al. [33], according to which females in group II division 1 malocclusion had significantly thicker STFP while in Class III there were no significant differences between genders. These differences may result from the selection of different landmarks used in the cephalometric analysis as well as different allocation of subjects to groups. Thicker STFP in males in comparison with females was demonstrated in two other studies, where Arnett’s cephalometric analysis was used [ [16, 34]; yet, both studies compared males and females with normal occlusion. Thicker STFP in males were also obtained from studies in which the cone-beam computed tomography (CBCT)[ [14, 35] and the magnetic resonance imaging (MRI) [23, 36] examinations were used. Domaracki et al. [37] conducted a study on human cadavers using a needle puncture and did not reveal any statistically significant differences in STFP thickness between males and females. This may be due to the examination method used in this study.

Studies of children aged 8–12 years conducted in Brasil by Pithon et al. [12] demonstrated that boys with the skeletal Class I maloclussion had significantly thicker soft tissues than girls at the base of the nose, in the subnasal and the upper lip areas. Lopatiene examined children aged 14–16 years and found higher values for the upper lip thickness in boys compared with girls [23]. Hoffelder reported, that the upper and lower lips were thicker in boys than in girls in most of the examined age groups [20]. Gibelli noted thicker STFP in boys versus girls, and the differences were higher in the oldest of the examined age groups (14–18 years) [18]. Sexual dimorphism in relation to STFP in children was also observed in other studies [19]. According to Jeelani, sexual dimorphism with regard to STFP thickness manifested itself at the age of 13 years [31]. Utsuno et al. [17] denied any manifestation of sexual dimorphism relative to STFT in children younger than 11 years; it can be observed after the age of 12 years.

### Limitations

Absence of data on body mass or the body mass index in the study group may be regarded as a limitation of this study. According to reports in the literature, BMI may significantly affect facial soft tissue thickness and therefore it should be considered [38, 39]. However, due to the retrospective character of this study and absence of routinely collected data on height and weight in adult patients, calculating BMI was not possible. Considering the large sample size in the present study, it can be assumed that the results represent a mean body masses in the Polish/ Caucasian populations. All the patients included in the study were Caucasians, which is another limitation of the study. The performed cephalometric analysis of the STFP was used to compare the thickness of soft tissues in specific areas between groups. It does not have specific norms serving as a reference point regarding the normal values of the STFP thickness at the different facial areas.

## Conclusions

The evaluation of the STFP thickness revealed the presence of sexual dimorphism. Males have higher STFP thickness than females, both in adult and in growing patients.

The study also demonstrated the presence of compensatory soft tissue adaptations to skeletal malocclusions, manifested as increased thickness of the STFP in the upper lip and nasolabial area in patients with skeletal Class III malocclusion, as well as increased thickness of the lower lip in patients with skeletal Class II malocclusion. The results of the study indicated increased thickness of the STFP in adults compared to children and adolescents, particularly within the male gender.

## Data Availability

The datasets used and analysed during the study are available from the corresponding author on the reasonable request.
